# Respiratory syncytial virus infection activates IL-13–producing group 2 innate lymphoid cells through thymic stromal lymphopoietin

**DOI:** 10.1016/j.jaci.2016.01.050

**Published:** 2016-09

**Authors:** Matthew T. Stier, Melissa H. Bloodworth, Shinji Toki, Dawn C. Newcomb, Kasia Goleniewska, Kelli L. Boyd, Marc Quitalig, Anne L. Hotard, Martin L. Moore, Tina V. Hartert, Baohua Zhou, Andrew N. McKenzie, R. Stokes Peebles

**Affiliations:** a Department of Pathology, Microbiology, and Immunology, Vanderbilt University School of Medicine, Nashville, Tenn; b Division of Allergy, Pulmonary and Critical Care Medicine, Department of Medicine, Vanderbilt University School of Medicine, Nashville, Tenn; c Division of Infectious Disease, Department of Pediatrics, Emory University School of Medicine, and Children's Healthcare of Atlanta, Atlanta, Ga; d Wells Center for Pediatric Research, Department of Pediatrics, Indiana University School of Medicine, Indianapolis, Ind; e MRC Laboratory of Molecular Biology, Cambridge University, Cambridge, United Kingdom

**Keywords:** Group 2 innate lymphoid cells, IL-13, IL-33, thymic stromal lymphopoietin, respiratory syncytial virus, type 2 immunity (T_H_2), BAL, Bronchoalveolar lavage, ILC, Innate lymphoid cell, ILC2, Group 2 innate lymphoid cell, INSPIRE, Infant Susceptibility to Pulmonary Infections and Asthma Following RSV Exposure, KO, Knockout, MFI, Mean fluorescence intensity, PAS, Periodic acid–Schiff, PFU, Plaque-forming units, RSV, Respiratory syncytial virus, TSLP, Thymic stromal lymphopoietin, TSLPR, Thymic stromal lymphopoietin receptor, WT, Wild-type

## Abstract

**Background:**

Respiratory syncytial virus (RSV) is a major health care burden with a particularly high worldwide morbidity and mortality rate among infants. Data suggest that severe RSV-associated illness is in part caused by immunopathology associated with a robust type 2 response.

**Objective:**

We sought to determine the capacity of RSV infection to stimulate group 2 innate lymphoid cells (ILC2s) and the associated mechanism in a murine model.

**Methods:**

Wild-type (WT) BALB/c, thymic stromal lymphopoietin receptor (TSLPR) knockout (KO), or WT mice receiving an anti-TSLP neutralizing antibody were infected with the RSV strain 01/2-20. During the first 4 to 6 days of infection, lungs were collected for evaluation of viral load, protein concentration, airway mucus, airway reactivity, or ILC2 numbers. Results were confirmed with 2 additional RSV clinical isolates, 12/11-19 and 12/12-6, with known human pathogenic potential.

**Results:**

RSV induced a 3-fold increase in the number of IL-13–producing ILC2s at day 4 after infection, with a concurrent increase in total lung IL-13 levels. Both thymic stromal lymphopoietin (TSLP) and IL-33 levels were increased 12 hours after infection. TSLPR KO mice did not mount an IL-13–producing ILC2 response to RSV infection. Additionally, neutralization of TSLP significantly attenuated the RSV-induced IL-13–producing ILC2 response. TSLPR KO mice displayed reduced lung IL-13 protein levels, decreased airway mucus and reactivity, attenuated weight loss, and similar viral loads as WT mice. Both 12/11-19 and 12/12-6 similarly induced IL-13–producing ILC2s through a TSLP-dependent mechanism.

**Conclusion:**

These data demonstrate that multiple pathogenic strains of RSV induce IL-13–producing ILC2 proliferation and activation through a TSLP-dependent mechanism in a murine model and suggest the potential therapeutic targeting of TSLP during severe RSV infection.

## Graphical abstract

**Figure:**
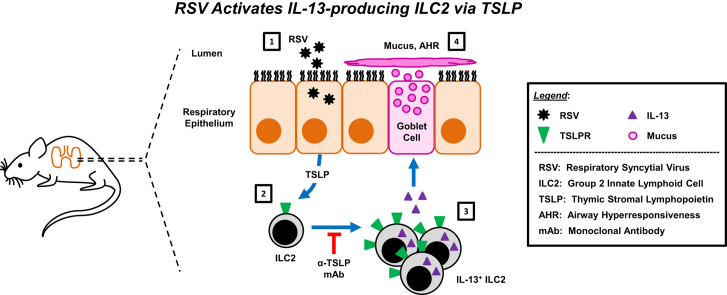

Respiratory syncytial virus (RSV) is the leading cause of infant hospitalization in the United States.^1^ RSV induces bronchiolitis and viral pneumonia and can lead to death in severe cases. Current therapeutic options are limited. Ribavirin, a nucleoside analog that inhibits viral replication, has shown poor effectiveness in treating RSV-induced disease.^2^ Corticosteroids have also proved ineffective during virus-induced bronchiolitis, failing to reduce the number of hospital admissions, length of stay, or disease severity.^3^, ^4^, ^5^ The majority of treatment strategies are supportive, focusing on fluid and respiratory maintenance. Widely available and cost-effective preventative options are also lacking. The only US Food and Drug Administration–approved preventative therapy is palivizumab, an antibody directed against the surface-exposed RSV fusion protein, which, when given as prophylaxis, decreases the number of RSV-associated hospitalizations by up to 55%.^6^, ^7^ Although effective, palivizumab is prohibitively expensive for widespread use and is currently only recommended for infants in the first year of life with chronic lung disease, with hemodynamically significant congenital heart disease, or born significantly premature (<29 weeks).^8^ Moreover, decades of research have failed to yield a safe and effective RSV vaccine.

Our incomplete understanding of the immune response to RSV presents a major obstacle to the development of new therapeutics and a vaccine. It is unclear which aspects of the immune response are protective and which are detrimental. Severe RSV infection in infants is characterized by airway epithelial cell destruction and sloughing, mucus production, peribronchiolar inflammation, and pulmonary obstruction.^9^ Classically, mucus production has been associated with activation of CD4^+^ T_H_2 cells.^10^ CD4^+^ T_H_2 cells mediate responses to certain viral, parasitic, bacterial, and allergen exposures and produce the cytokine IL-13, a central mediator of airway reactivity and mucus production.^11^ However, no studies have evaluated early IL-13 production during RSV infection before CD4^+^ T_H_2 cell maturation. A better understanding of the host immune response to RSV will inform rational design of new clinical and pharmacologic interventions.

Group 2 innate lymphoid cells (ILC2s) are an early and significant source of IL-13 in several other pulmonary diseases.^12^, ^13^, ^14^, ^15^, ^16^, ^17^, ^18^, ^19^, ^20^, ^21^ ILC2s are temporally and functionally distinct from T_H_2 cells. Unlike T and B cells, ILC2s do not have specific antigen receptors. Rather, ILC2s act primarily as early innate effector cells that respond directly to cytokine stimulation.^22^, ^23^, ^24^ Specifically, the epithelium-associated cytokines IL-25, IL-33, and thymic stromal lymphopoietin (TSLP) induce ILC2 proliferation and activation.^12^, ^22^, ^23^, ^24^ Lung-resident ILC2s express high levels of IL-5 and IL-13 but minimal IL-4.^25^ This is in contrast to mature effector T_H_2 cells, which express all 3 cytokines. Comparatively, early reports suggest that ILC2s are significantly more potent than T_H_2 cells, elaborating greater than 10 times the amount of T_H_2 cytokines on a per-cell basis.^25^ Our understanding of lung ILC2s has largely come from investigations into their role in patients with allergic asthma, where they have been characterized as important mediators of airway responsiveness, eosinophilia, and mucus production.^12^, ^14^, ^18^, ^19^, ^20^ Several studies have also shown that ILC2s can play a role in pathologic changes during influenza and rhinovirus infections through either IL-33 or IL-25, although the effect of TSLP on virus-induced ILC2 activation in the lungs remains unknown.^15^, ^16^, ^17^, ^21^, ^26^

We hypothesized that RSV induces a robust IL-13–producing ILC2 response during the early phase of RSV infection in a murine model. To test this hypothesis, we used the RSV clinical strain 01/2-20, which was isolated from a patient with RSV-induced bronchiolitis and potentiated IL-13 production in mice.^27^ We identified a significant increase in the total lung IL-13 concentration and the number of IL-13^+^ ILC2s at day 4 after infection. TSLP signaling was required for this ILC2 enhancement. Moreover, RSV-infected thymic stromal lymphopoietin receptor (TSLPR)–deficient mice had reduced lung IL-13 protein concentration and decreased airway mucus and reactivity, were partially protected from RSV-induced weight loss, and had comparable viral loads to wild-type (WT) mice. Finally, infection of mice with recent clinical isolates of RSV with known human pathogenic potential demonstrated a similar induction of IL-13^+^ ILC2s at day 4 after infection that required TSLP. Collectively, these data demonstrate a critical role for TSLP in RSV-induced ILC2 activation, suggesting this cytokine as a potential therapeutic target to treat the immune-associated pathology of severe RSV-associated illness.

## Methods

Full methods are available in the Methods section in this article's Online Repository at www.jacionline.org; a brief summary is provided here.

### Viruses and mice

RSV strains 01/2-20,^27^ which was isolated from a patient in 2001 in the Vanderbilt Vaccine Clinic, and 12/11-19 and 12/12-6,^28^ which were isolated in 2012 from hospitalized patients with RSV-induced lower respiratory tract infection as part of the Infant Susceptibility to Pulmonary Infections and Asthma Following RSV Exposure (INSPIRE) study, were propagated and titrated in HEp-2 cells, as previously described.^29^ Female 8- to 12-week-old IL-33 citrine reporter (IL-33-deficient; IL-33 knockout [KO]) mice, TSLPR-deficient (TSLPR KO) mice, or WT BALB/c mice were purchased from the Jackson Laboratory (Bar Harbor, Me). Mice were housed in microisolator cages under specific pathogen-free conditions. For infection, mice were anesthetized with a ketamine/xylazine solution and inoculated by means of intranasal delivery of 3.0 × 10^6^ plaque-forming units (PFU) of RSV 01/2-20, 1.0 × 10^6^ PFU of RSV 12/11-19, 9.0 × 10^5^ PFU of RSV 12/12-6, or an equal volume of mock inoculum, as previously described.^29^

### ELISA

Protein measurements for IL-25, IL-33, TSLP, IL-4, IL-5, and IL-13 (R&D Systems, Minneapolis, Minn) were performed on frozen and mechanically disrupted lungs, according to the manufacturer's instructions.

### Flow cytometry

Lungs were digested in RPMI media with 5% FBS, 1 mg/mL collagenase, and 0.02 mg/mL DNase I for 60 minutes at 37°C. A single-cell suspension was generated by straining these digestions through a 70-μm filter. RBC lysis (BioLegend, San Diego, Calif) was performed, according to the manufacturer's instructions. Cells were restimulated with 10 ng/mL phorbol 12-myristate 13-acetate and 1 μmol/L ionomycin in the presence of 0.07% monensin in Iscove modified Dulbecco medium supplemented with 10% FBS, 0.01 mmol/L nonessential amino acids, penicillin/streptomycin, and 1 mmol/L sodium pyruvate for 6 hours at 37°C. Cells were stained for viability and cell-surface proteins, fixed/permeabilized, and stained for intracellular antigens. All samples were run on a BD LSR II Flow Cytometer (BD Biosciences, San Jose, Calif) and analyzed with FlowJo software (Version 10; FlowJo, Ashland, Ore). Total innate lymphoid cells (ILCs) were defined as Lin^−^CD45^+^CD25^+^CD127^+^ cells, where Lin includes CD3, CD5, CD45R (B220), CD11b, Gr-1 (Ly-6G/C), 7-4, and Ter-119. ILC2s were defined as ILCs that expressed IL-5, IL-13, or both. T cells were defined as CD45^+^CD3^+^ cells. Mean fluorescence intensity (MFI) was determined as the geometric mean.

### *In vivo* TSLP neutralization

At either 6 or 36 hours after RSV infection, mice received a single dose of 200 μg of 28F12, an anti-TSLP mAb with established *in vivo* neutralizing capacity,^30^, ^31^, ^32^ or isotype control antibody through intraperitoneal injection.

### Periodic acid–Schiff staining

Lungs were fixed in 10% neutral buffered formalin. Fixed lungs were paraffin embedded, sectioned (5 μm), and stained with periodic acid–Schiff (PAS) to visualize mucus, as previously described.^33^ Small- and medium-sized airways were scored for mucus by a trained pathologist blind to the experimental information.

### Airway reactivity

Airway reactivity was measured, as previously described.^34^, ^35^

### Statistical analysis

Data were analyzed with GraphPad Prism software (version 5; GraphPad Software, La Jolla, Calif). Differences between groups were evaluated by using the unpaired *t* test, 1-way ANOVA with the Bonferroni posttest, or 2-way ANOVA with the Dunn multiple comparison test, as appropriate. Measurements of less than the limit of detection were assigned half of the value of the limit of detection to allow for statistical analyses.

## Results

### RSV infection increases the concentration of IL-13 and the number of IL-13–producing ILC2s in the lungs at day 4 after infection

**Fig E1:**
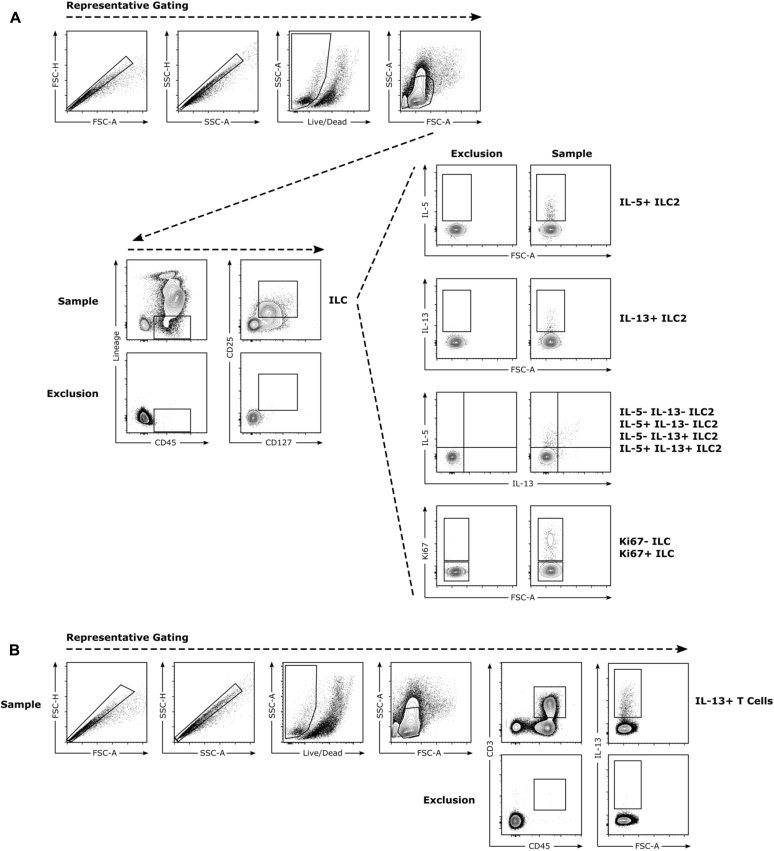
Flow cytometric gating strategies. **A,** ILCs were defined as viable Lin^−^CD45^+^CD25^+^CD127^+^ cells. ILC2s were defined as ILCs that were positive for either IL-5 or IL-13. **B,** T cells were defined as viable CD45^+^CD3^+^ cells. *FSC*, Forward scatter; *SSC*, side scatter.

**Fig 1:**
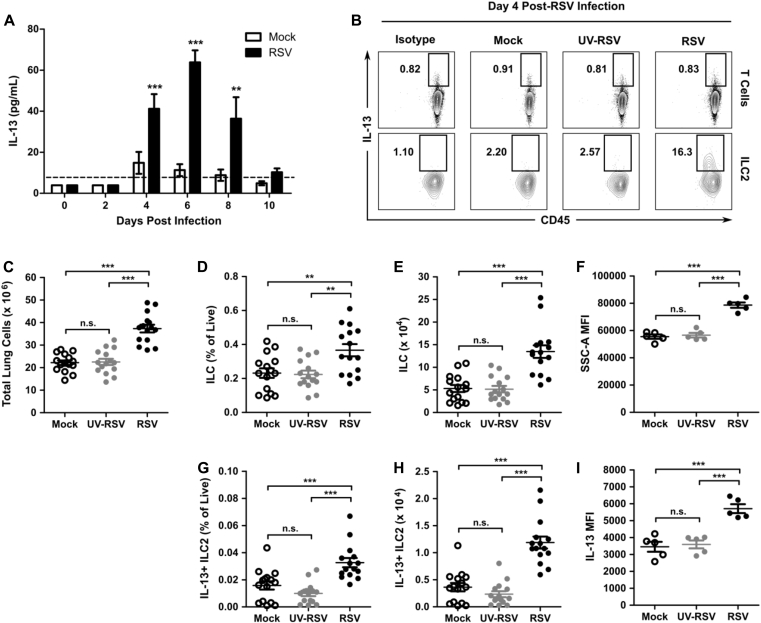
RSV infection induced whole-lung IL-13 accumulation and IL-13–producing ILC2s at day 4 after infection. WT mice were infected with 3 × 10^6^ PFU of RSV strain 01/2-20 and harvested on days 0 to 10 after infection. **A,** ELISA for IL-13 in whole-lung homogenate (right lung only). **B,** Representative IL-13 expression measured by using flow cytometry in ILC2s (Lin^−^CD45^+^CD25^+^CD127^+^IL-13^+^) and T cells (CD45^+^CD3^+^IL-13^+^) compared with isotype control staining at day 4 after infection. Numbers indicate the percentage of T cells or ILC2s within the gated region. **C,** Total number of live cells. **D,** Percentage of live cells that are ILCs. **E,** Total number of ILCs. **F,** MFI of side-scatter area in ILCs. **G,** Percentage of live cells that are IL-13^+^ ILC2s. **H,** Total numbers of IL-13^+^ ILC2s. **I,** MFI of IL-13 in IL-13^+^ ILC2s measured at day 4 after infection. Data are plotted as means ± SEMs. For Fig 1, *A*, n = 8 to 14 mice per group combined from 3 independent experiments. For Fig 1, *B*, *F*, and *I*, data are representative of 3 independent experiments. For Fig 1, *C-E* and *G-H*, n = 15 mice per group combined from 3 independent experiments. **P < .01 and ***P < .001, 1-way (Fig 1, *C-F*) or 2-way (Fig 1, *A*) ANOVA. *n.s.*, Not significant. The *dashed line* is the limit of detection of the assay.

We first determined the kinetics of IL-13 expression in the lungs of RSV-infected mice. Eight-week-old WT mice were infected with 3 × 10^6^ PFU of RSV clinical isolate 01/2-20, and lungs were harvested on days 0, 2, 4, 6, 8, and 10 for measurement of IL-13 by using ELISA. There was a significant induction of IL-13 protein in the lungs of RSV-infected mice compared with levels seen in mock-infected mice beginning at day 4 after infection and continuing through day 8 after infection (Fig 1, *A*). We hypothesized that ILC2s rather than T cells were the predominant source of the early IL-13 observed at day 4 after infection because this time point precedes the adaptive immune response during RSV infection.^36^ No unique surface markers are presently known to identify ILC2s exclusively. We defined ILCs as hematopoietic lineage marker (CD3, CD5, B220, CD11b, Gr-1, 7-4, and Ter-119)–negative CD45^+^, CD25^+^, and CD127^+^ and ILC2s as ILCs that are IL-5^+^, IL-13^+^, or both by using flow cytometry (see Fig E1 in this article's Online Repository at www.jacionline.org).^37^ At day 4 after infection, we noted a significant increase in the total number of cells in the lung, the percentage of live cells that were ILCs and IL-13^+^ ILC2s, and the total number of ILCs and IL-13^+^ ILC2s in RSV-infected mice relative to mock-infected mice (Fig 1, *B-E* and *G-H*). Moreover, viral replication was required for this phenomenon because inoculation with UV-inactivated virus did not increase the number of ILCs or IL-13^+^ ILC2s compared with mock inoculum (Fig 1, *B-E* and *G-H*). Additionally, the ILC compartment had an increased side-scatter MFI, which is consistent with an activate state (Fig 1, *F*).^18^, ^38^ Finally, the MFI of IL-13 in ILC2s was higher in the RSV-infected group compared with that seen after inoculation with mock preparation or UV-inactivated virus, suggesting increased production of IL-13 on a per-ILC2 basis after RSV infection (Fig 1, *I*). As expected at day 4 after infection, IL-13^+^ T cells were not detected because staining for IL-13 in the CD3^+^ T-cell compartment was comparable with isotype signal (Fig 1, *B*).

**Fig E2:**
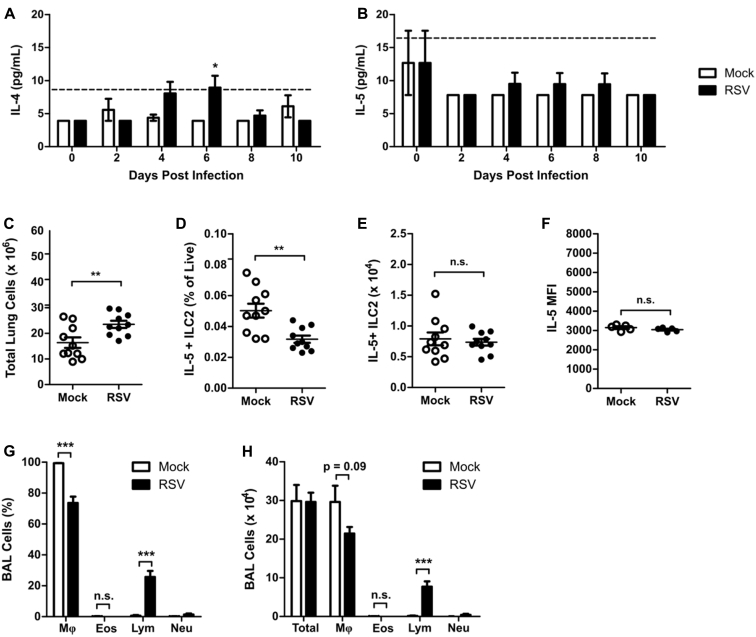
RSV does not induce IL-5–producing ILC2s. WT mice were infected with 3 × 10^6^ PFU of RSV strain 01/2-20 and harvested on days 0 to 10 after infection. **A** and **B,** ELISA for IL-4 (Fig E2, *A*) and IL-5 (Fig E2, *B*) in whole-lung homogenate. **C,** Total number of live cells in the lungs at day 4 after infection. **D,** IL-5^+^ ILC2s as a percentage of viable cells as measured by using flow cytometry at day 4 after infection. **E,** Total number of IL-5^+^ ILC2s as measured by using flow cytometry at day 4 after infection. **F,** MFI of IL-5 staining in ILC2s as measured by using flow cytometry at day 4 after infection. **G,** Percentage of cells in BAL fluid at day 4 after infection. **H,** Total number of cells in BAL fluid at day 4 after infection. Data are plotted as means ± SEMs. For Fig E2, *A* and *B*, n = 8-14 mice per group combined from 2 independent experiments. For Fig E2, *C-E*, *G*, and *H*, n = 10 mice per group combined from 2 independent experiments. For Fig E2, *F*, n = 5 mice per group representative of 2 independent experiments. *P < .05, **P < .01, and ***P < .001, unpaired *t* test (Fig E2, *C-H*) or 2-way ANOVA (Fig E2, *A* and *B*). *n.s.*, Not significant. The *dashed line* is the limit of detection of the assay. *Eos*, Eosinophils; *Lym*, lymphocytes; *Mφ*, macrophages; *Neu*, neutrophils.

ILC2s also have the potential to express considerable amounts of IL-5 in addition to IL-13. We did not identify appreciable concentrations of IL-4 or IL-5 by using ELISA in RSV-infected mice compared with mock-infected mice during the first 10 days after RSV infection, with only IL-4 concentrations being statistically significant but just slightly greater than the limit of detection at day 6 after infection (see Fig E2, *A* and *B*, in this article's Online Repository at www.jacionline.org). Consistent with this finding, we did not identify a significant difference in the total number of IL-5^+^ ILC2s or in MFI of IL-5 at day 4 after infection between mock- and RSV-infected mice (see Fig E2, *C-F*). Furthermore, RSV infection did not increase the percentage or total number of eosinophils in bronchoalveolar lavage (BAL) fluid at day 4 after infection compared with values in mock-infected mice (see Fig E2, *G-H*). These data suggest that the predominant functional capacity of ILC2s induced during RSV infection is the expression of IL-13.

**Fig 2:**
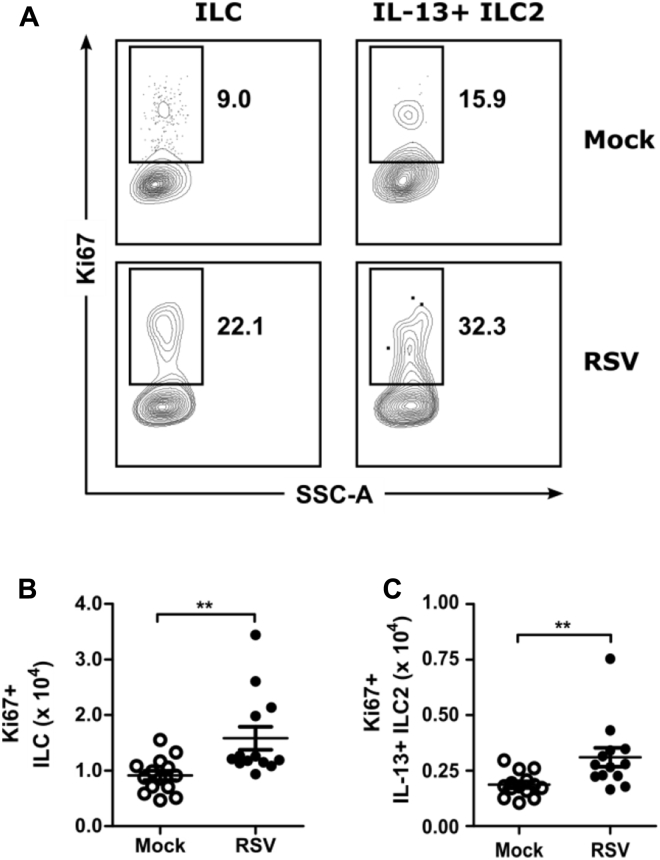
RSV-stimulated ILC2 proliferation at day 4 after infection. WT mice were infected with 3 × 10^6^ PFU of RSV strain 01/2-20, and lungs were harvested for flow cytometry at day 4 after infection. Cells were gated for viable IL-13^+^ ILC2s and analyzed for Ki67. **A,** Representative plots for Ki67 staining. Numbers indicate the percentage of Ki67^+^ ILCs or Ki67^+^IL-13^+^ ILC2s within the gated region. **B** and **C,** Total number of Ki67^+^ ILCs (Fig 2, *B*) and Ki67^+^IL-13^+^ ILC2s (Fig 2, *C*). Data are plotted as means ± SEMs. N = 13-14 mice per group combined from 2 independent experiments. **P < .01, unpaired *t* test.

Moreover, there was a significant increase in the number of total ILCs and IL-13^+^ ILC2s that stained for Ki67, a marker of cellular proliferation, at day 4 after infection in the RSV-infected group compared with the mock-infected group (Fig 2). These data suggest that local proliferation of ILC2s within the lungs contributes to the increase in the total number of ILCs and IL-13^+^ ILC2s as a result of RSV infection. Collectively, these data suggest that ILC2s are a source of early IL-13 production after RSV infection.

### TSLP is necessary for the RSV-induced ILC2 response

We next sought to understand the mechanism by which RSV drives ILC2 accumulation in the lungs. TSLP has not previously been recognized to affect ILC2s during viral respiratory tract infection; however, it is a known stimulus of ILC2s in other disease models and can be released from epithelial cells in a fashion to IL-33 and IL-25. Moreover, previous studies have shown that infections with RSV strains A2 and Line 19 provoke TSLP expression in murine lungs, although these studies focused on the effect of TSLP on dendritic cells and T_H_2 cells.^39^, ^40^

**Fig 3:**
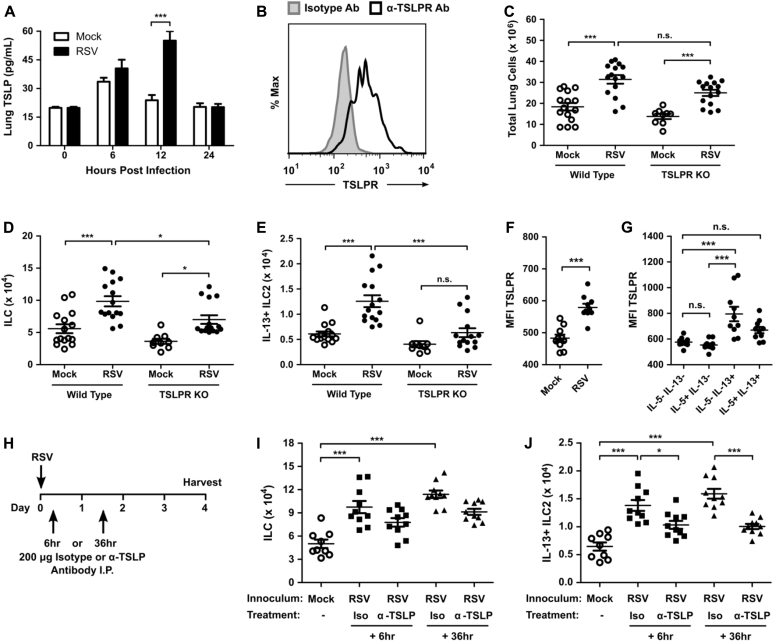
RSV-induced TSLP signaling is required for ILC2 activation. WT or TSLPR KO mice were infected with 3 × 10^6^ PFU of RSV strain 01/2-20, and lungs were harvested for ELISA or flow cytometry. **A,** ELISA for TSLP in the whole-lung homogenate. **B,** TSLPR expression determined by using flow cytometry in IL-13^+^ ILC2s from naive mice. **C-E,** Total number of live cells (Fig 3, *C*), ILCs (Fig 3, *D*), and IL-13^+^ ILC2s (Fig 3, *E*) as measured by using flow cytometry at day 4 after infection. **F,** MFI of TSLPR on ILCs from mock- and RSV-infected mice. **G,** MFI of TSLPR on ILC subsets in RSV-infected mice. **H,** Protocol for *in vivo* neutralization of TSLP. *I.P.*, Intraperitoneal. **I** and **J,** Total number of ILCs (Fig 3, *I*) and IL-13^+^ ILC2s (Fig 3, *J*), as measured by using flow cytometry at day 4 after infection. Data are plotted as means ± SEMs. For Fig 3, *A*, n = 5 to 10 mice per group combined from 2 independent experiments. For Fig 3, *B*, data are representative of 2 independent experiments. For Fig 3, *C-E*, n = 9-15 mice per group combined from 3 independent experiments. For Fig 3, *F* and *G*, n = 10 mice per group. For Fig 3, *I* and *J*, n = 9-10 mice per group combined from 2 independent experiments. *P < .05 and ***P < .001, unpaired *t* test (Fig 3, *F*), 1-way ANOVA (Fig 3, *C-E*, *G*, *I*, and *J*), or 2-way ANOVA (Fig 3, *A*). *n.s.*, Not significant.

To determine whether TSLP is required for the RSV-induced ILC2 response, we first measured the concentration of TSLP using ELISA in the lungs of mock- and RSV-infected mice. We identified a significant increase in the total concentration of TSLP in the lungs of RSV-infected mice compared with mock-infected mice at 12 hours after infection (Fig 3, *A*). Measurements of TSLP levels by using ELISA between 24 and 96 hours after infection were all less than the limit of detection (data not shown).

**Fig E3:**
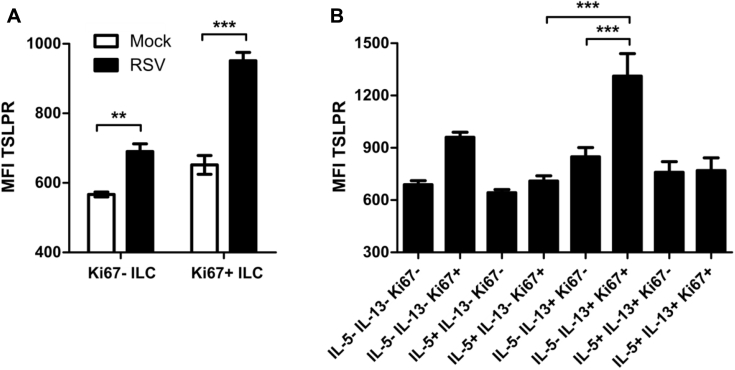
TSLPR expression in Ki67^+^ and Ki67^−^ subsets of ILCs at day 4 after RSV infection. WT mice were infected with 3 × 10^6^ PFU of RSV strain 01/2-20, and lungs were harvested for flow cytometry. **A,** MFI of TSLPR expression determined by means of flow cytometry at day 4 after RSV infection stratified by Ki67 expression status. **B,** Subset analysis of ILCs stratified by Ki67 expression status in RSV-infected mice at day 4 after infection. Data are plotted as means ± SEMs. For Fig E3, *A* and *B*, n = 5 mice per group. **P < .01, and ***P < .001, 1-way ANOVA.

Consistent with a role for TSLP in lung ILC2 activation during RSV infection, we identified that lung ILC2s in naive mice express TSLPR and are thus poised to respond directly to TSLP (Fig 3, *B*). To determine the role of TSLP during RSV-induced ILC2 activation, we assayed the total number of ILCs and IL-13^+^ ILC2s in TSLPR KO mice at day 4 after infection. Both WT and TSLPR KO mice displayed a substantial inflammatory response to RSV infection (Fig 3, *C*). However, RSV-infected TSLPR KO mice had significantly reduced numbers of total ILCs compared with WT RSV-infected mice (Fig 3, *D*). Among these ILCs, RSV-infected TSLPR KO mice had similar numbers of IL-13^+^ ILC2s compared with mock-infected TSLPR KO mice and significantly reduced numbers of IL-13^+^ ILC2s compared with RSV-infected WT mice (Fig 3, *E*). TSLPR expression was also upregulated on total ILCs after RSV infection (Fig 3, *F*) and was selectively enhanced on IL-5^−^IL-13^+^ ILC2s (Fig 3, *G*). Similarly, TSLPR expression was upregulated on Ki67^+^ ILCs compared with Ki67^−^ ILCs (see Fig E3, *A*, in this article's Online Repository at www.jacionline.org), and high levels of expression were restricted to Ki67^+^IL-5^−^IL-13^+^ ILC2s (see Fig E3, *B*).

To determine the plausibility of exogenously targeting TSLP signaling during RSV infection, we tested the effect of a TSLP neutralizing antibody (clone 28F12) on RSV-driven ILC2 induction (Fig 3, *H*). RSV-infected mice treated either 6 or 36 hours after infection with a single 200-μg dose of TSLP neutralizing antibody had a trend for a decrease in the total numbers of ILCs compared with those seen in RSV-infected mice treated with isotype control antibody, but this was not statistically significant (Fig 3, *I*). Importantly, there was a statistically significant decrease in the number of IL-13^+^ ILC2s in the groups receiving TSLP neutralizing antibody at either 6 or 36 hours after infection compared with RSV-infected mice treated with isotype control antibody (Fig 3, *J*).

**Fig E4:**
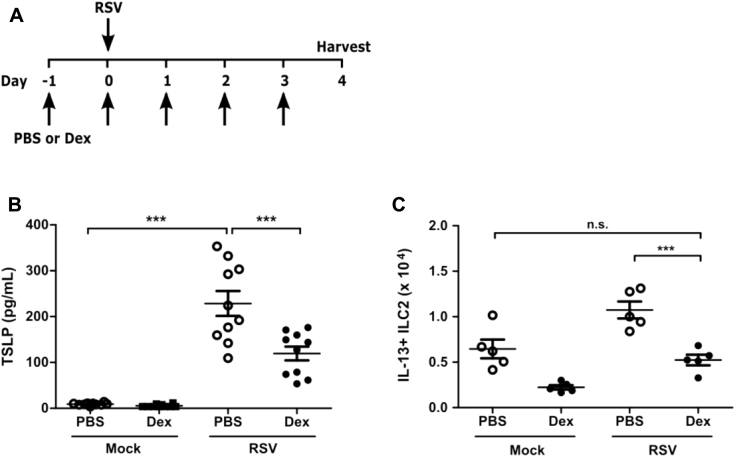
Pretreatment of mice with dexamethasone *(Dex)* decreases TSLP expression and ILC2 activation. WT mice were treated every 24 hours with 2.5 mg/kg dexamethasone beginning 1 day before infection until harvest. Mice were infected on day 0 with 3 × 10^6^ PFU of RSV strain 01/2-20, and lungs were harvested for flow cytometry at day 4 after infection. **A,** Protocol for dexamethasone administration. **B,** ELISA for TSLP in the whole-lung homogenate at 12 hours after infection. **C,** Total numbers of IL-13^+^ ILC2s as measured by using flow cytometry at day 4 after infection. Data are plotted as means ± SEMs. For Fig E4, *B*, n = 10 mice per group combined from 2 independent experiments. For Fig E4, *C*, n = 5 mice per group representative of 3 similar experiments. ***P < .001, 1-way ANOVA. *n.s.*, Not significant.

Consistent with these data, we found that treatment of mice every 24 hours with dexamethasone beginning 24 hours before infection decreased lung TSLP expression and the total number of IL-13^+^ ILC2s at day 4 after infection compared with vehicle-treated mice, further highlighting the importance of TSLP during RSV-induced ILC2 activation (see Fig E4 in this article's Online Repository at www.jacionline.org). Of note, dexamethasone was administered prophylactically in our model, a key distinction from human trials of corticosteroids in which administration has been after infection and demonstrated to be ineffective. Collectively, these data strongly suggest a TSLP-dependent mechanism for the induction of IL-13–producing ILC2s after RSV infection that could be potentially exploited therapeutically.

**Fig E5:**
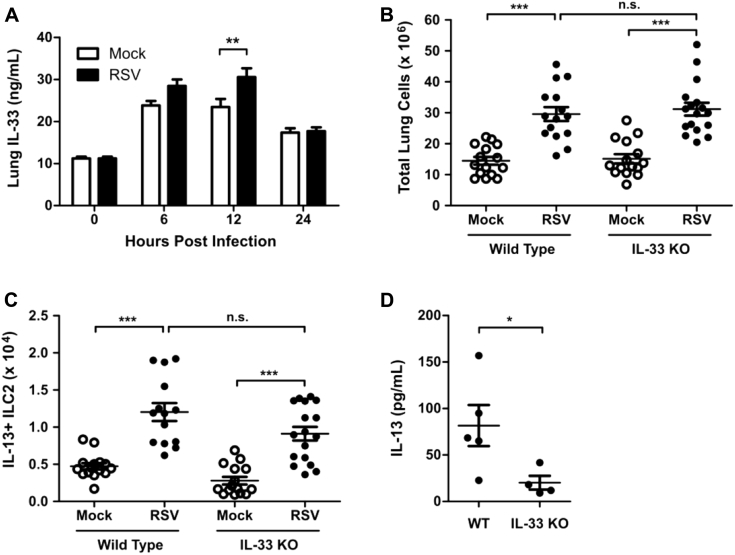
RSV-induced IL-33 and ILC2s. WT or IL-33 KO mice were infected with 3 × 10^6^ PFU of RSV strain 01/2-20, and lungs were harvested for ELISA or flow cytometry. **A,** ELISA for IL-33 in the whole-lung homogenate. **B,** Total number of live cells in the lungs at day 4 after infection. **C,** Total numbers of IL-13^+^ ILC2s as measured by using flow cytometry at day 4 after infection. **D,** ELISA for IL-13 in the whole-lung homogenate (right and left lung) at day 4 after infection. Data are plotted as means ± SEMs. For Fig E5, *A*, n = 5-10 mice per group combined from 2 independent experiments. For Fig E5, *B* and *C*, n = 13-17 mice per group combined from 3 independent experiments. For Fig E5, *D*, n = 4-5 mice per group from 1 independent experiment. *P < .05, **P < .01, and ***P < .001, 1-way (Fig E5, *B* and *C*) or 2-way (Fig E5, *A*) ANOVA. *n.s.*, Not significant.

IL-33 and IL-25 have been implicated as activators of ILC2s during infection with other respiratory tract viruses,^15^, ^16^, ^17^, ^21^ and RSV strain Line 19 has been shown to induce transcription of IL-25.^41^ To determine the importance of these cytokines during RSV infection, we measured the whole-lung concentration of IL-33 and IL-25 protein by using ELISA after RSV infection. We identified a significant increase in lung IL-33 protein levels in RSV-infected mice compared with those in mock-infected mice at 12 hours after infection (see Fig E5, *A*, in this article's Online Repository at www.jacionline.org). IL-25 levels were less than the limit of detection, as determined by using ELISA, across the first 96 hours after infection (data not shown). To determine whether IL-33 was required for activating ILC2s during RSV infection, we measured the total number of IL-13^+^ ILC2s in the lungs at day 4 after infection in WT and IL-33 KO mice. Both WT and IL-33 KO mice showed a significant inflammatory response to RSV, as measured by total lung cell numbers, compared with those after mock infection (see Fig E5, *B*). RSV infection induced a significant increase in numbers of IL-13^+^ ILC2s compared with those after mock infection in both WT and IL-33 KO mice, and there was no statistically significant difference in the total number of IL-13^+^ ILC2s between RSV-infected WT and IL-33 KO mice (see Fig E5, *C*). However, there was a significant decrease in the total lung concentration of IL-13 in the RSV-infected IL-33 KO mice compared with RSV-infected WT mice (Fig E5, *D*), highlighting an equivocal role for IL-33 during RSV-induced ILC2 activation in our murine model of infection.

### TSLPR-deficient mice exhibited decreased lung IL-13 levels, airway mucus, airway reactivity, and weight loss without an effect on viral load after RSV infection

**Fig E6:**
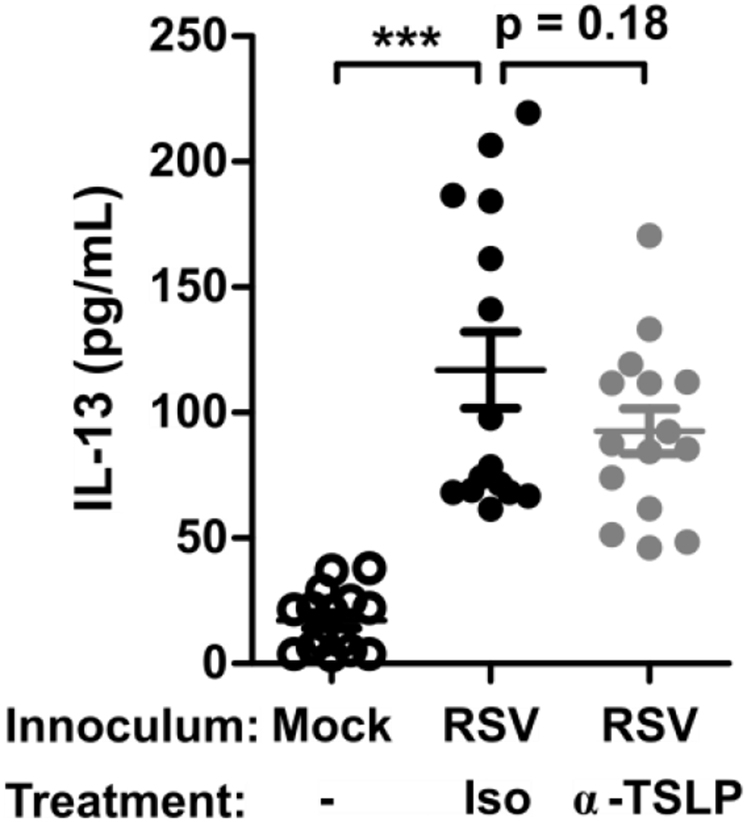
Lung IL-13 with TSLP neutralization. WT mice were infected with 3 × 10^6^ PFU of RSV strain 01/2-20 and treated with 200 μg of anti-TSLP antibody or isotype control at 6 hours after infection, and lungs were harvested for ELISA at day 4 after infection. ELISA data are shown for measurements of IL-13 levels in the whole-lung homogenate (right and left lung). Data are plotted as means ± SEMs. For Fig E6, *A*, n = 15 mice per group combined from 3 independent experiments. ***P < .001, 1-way ANOVA.

**Fig E7:**
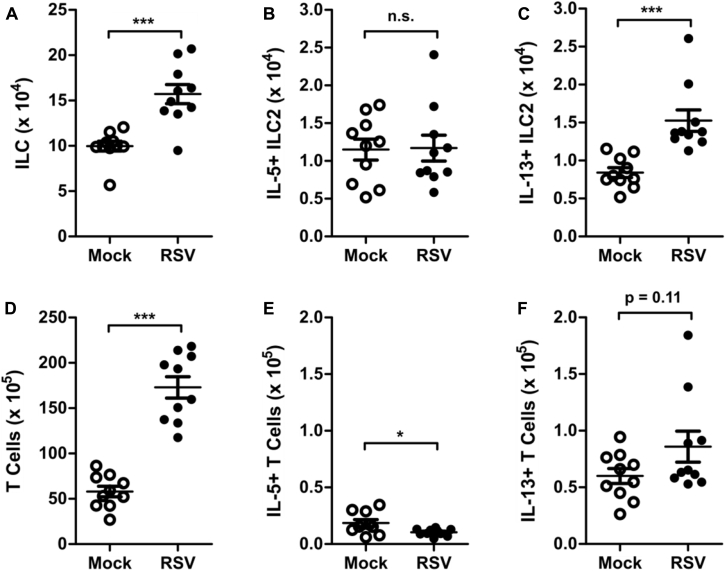
Analysis of ILCs and T cells at day 6 after infection. WT mice were infected with 3 × 10^6^ PFU of RSV strain 01/2-20 and harvested on day 6 after infection for flow cytometry. Total numbers of ILCs **(A)**, IL-5^+^ ILC2s **(B)**, IL-13^+^ ILC2s **(C)**, T cells **(D)**, IL-5^+^ T cells **(E)**, and IL-13^+^ T cells **(F)** were determined. Data are plotted as means ± SEMs. For Fig E6, *A-E*, n = 10 mice per group from 2 independent experiments. *P < .05 and ***P < .001, unpaired *t* test. *n.s.*, Not significant.

**Fig E8:**
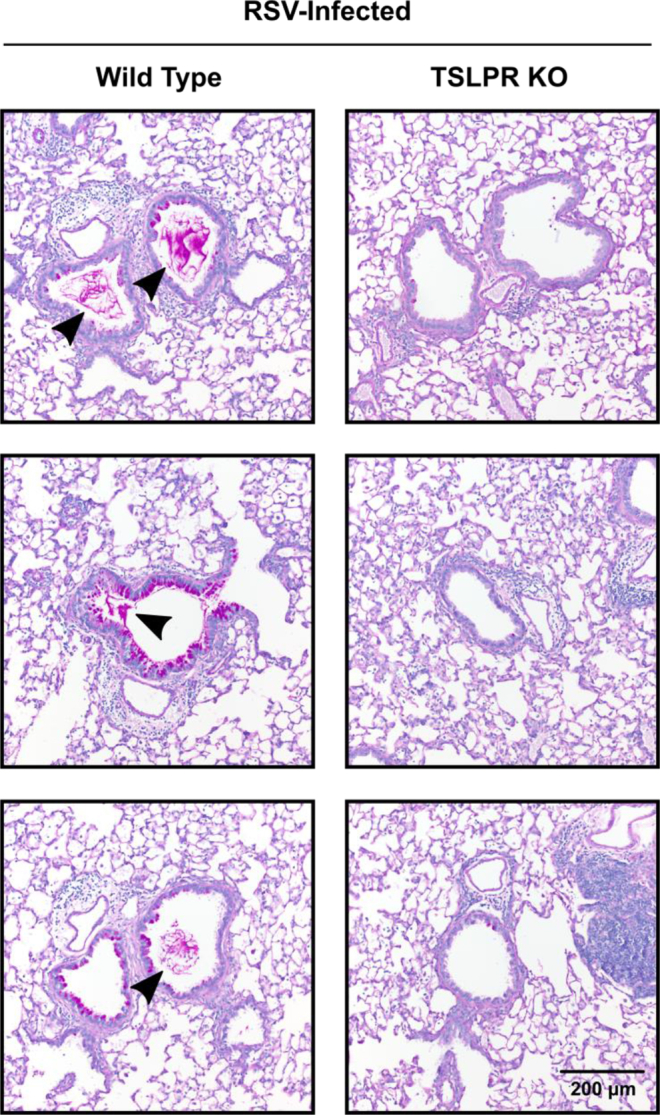
Supplementary histopathologic sections from WT and TSLPR-deficient mice at day 6 after infection. WT or TSLPR KO mice were infected with 3 × 10^6^ PFU of RSV strain 01/2-20. Additional representative PAS-stained sections of mucus-containing airways in the lungs on day 6 after infection (×20 magnification). The *arrowhead* denotes intraluminal mucus plugging.

**Fig 4:**
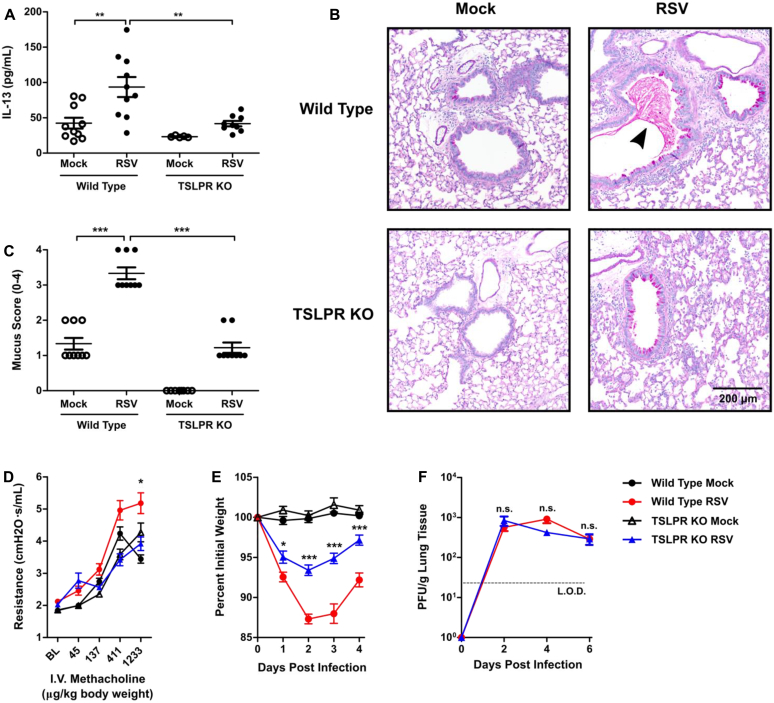
TSLPR deficiency attenuated RSV-induced IL-13 production, airway mucus accumulation, airway reactivity, and weight loss without negative effects on viral load. WT or TSLPR KO mice were infected with 3 × 10^6^ PFU of RSV strain 01/2-20. **A,** IL-13 levels measured by using ELISA from whole-lung homogenate (right and left lung) at day 4 after infection. **B,** Representative PAS-stained sections of mucus-containing airways in the lungs on day 6 after infection (×20 magnification). The *arrowhead* denotes intraluminal mucus plugging. **C,** Quantification of airway mucus from the experiment in Fig 4, *B*. Each *dot* represents a combined airway mucus score for an individual mouse. **D,** Airway reactivity measured at baseline and increasing doses of methacholine at day 6 after infection. *I.V.*, Intravenous. **E,** Daily weight loss displayed as a percentage of original body weight before infection. **F,** Lung viral titers determined at days 2, 4, and 6 after infection by using a plaque assay. For Fig 4, *A*, n = 5-10 mice combined from 2 independent experiments. For Fig 4, *B* and *C*, n = 8-9 mice per group combined from 2 independent experiments. For Fig 4, *D*, n = 5-10 mice per group. For Fig 4, *E*, n = 12-15 mice per group combined from 3 independent experiments. For Fig 4, *F*, n = 6-8 mice per group combined from 2 independent experiments. All data are plotted as means ± SEMs. *P < .05, **P < .01, and ***P < .001, 1-way (Fig 4, *A* and *C*) or 2-way (Fig 4, *D-F*) ANOVA. *n.s.*, Not significant. For Fig 4, *D-F*, statistical comparisons indicated are between the WT RSV and TSLPR KO RSV groups. The *dashed line* is the limit of detection of the assay. *BL*, Baseline.

To consider the efficacy of targeting TSLP for the attenuation of ILC2 responses during RSV infection, we considered the effect of TSLPR deficiency on RSV disease severity. Notably, RSV-infected TSLPR KO mice had significantly decreased levels of whole-lung IL-13 compared with RSV-infected WT mice at day 4 after infection (Fig 4, *A*). TSLP neutralization also had a trend for a decrease in IL-13 whole-lung concentrations at day 4 after infection, although this was not statistically significant (see Fig E6 in this article's Online Repository at www.jacionline.org). We next sought to determine the physiologic effect of early RSV-induced IL-13 on mucous cell metaplasia and airway mucus accumulation. To allow time for this early IL-13 to induce physiologic changes in the airways, we evaluated PAS-stained sections of lungs from mock- and RSV-infected WT and TSLPR KO mice at day 6 after infection. Importantly, at day 6 after infection, there remained a significant increase in the total number of ILCs, as well as IL-13^+^ ILC2s, but not IL-5^+^ ILC2s (see Fig E7, *A-C*, in this article's Online Repository at www.jacionline.org). Additionally, there was an increase in total numbers of CD3^+^ T cells but no significant increase in numbers of IL-5^+^ or IL-13^+^ T cells, although a trend for an increase was seen with IL-13^+^ T cells (see Fig E7, *D-F*). These data suggest that ILC2s remain a major component of the type 2 immune response at day 6 after infection. In mock-infected WT and TSLPR KO mice, there was minimal or absent mucous cell metaplasia (Fig 4, *B*). In RSV-infected WT mice we identified moderate mucous cell metaplasia and significant airway mucus accumulation with both intraluminal mucus strands and overt mucous plugging. RSV-infected TSLPR KO mice also exhibited moderate mucous cell metaplasia, but they had no substantial intraluminal mucus accumulation. Collective scoring of airways from multiple mice showed a significant decrease in mucus severity scores in RSV-infected TSLPR KO mice compared with RSV-infected WT mice (Fig 4, *C*, and see Fig E8 in this article's Online Repository at www.jacionline.org). The primary difference between these groups of mice was related to the amount of intraluminal obstructing mucus, which was never observed in the RSV-infected TSLPR KO mice.

To evaluate airway obstruction and reactivity, we performed a methacholine challenge experiment at day 6 after infection on mock- or RSV-infected WT and TSLPR KO mice. We saw a significant increase in airway reactivity in the RSV-infected WT group compared with the RSV-infected TSLPR KO mice, with increasing methacholine concentrations (Fig 4, *D*). No significant differences were observed between mock- and RSV-infected TSLPR KO mice.

Within the first 4 days after infection, both RSV-infected WT and TSLPR KO mice had significant weight loss relative to mock-infected control mice. However, RSV-infected TSLPR KO mice had significantly less weight loss compared with RSV-infected WT mice (Fig 4, *E*). Next, we sought to determine whether TSLPR deficiency altered viral load. We did not observe any significant differences in viral load at days 2, 4, or 6 after RSV infection between TSLPR KO and WT mice (Fig 4, *F*). Together, these data suggest that the absence of TSLP signaling is unlikely to exacerbate disease or increase viral load and implicate TSLP as a potential therapeutic target for attenuating ILC2-associated immunopathology during RSV infection.

### Multiple pathogenic clinical isolates of RSV induce IL-13–producing ILC2s through a TSLP-dependent mechanism

**Fig E9:**
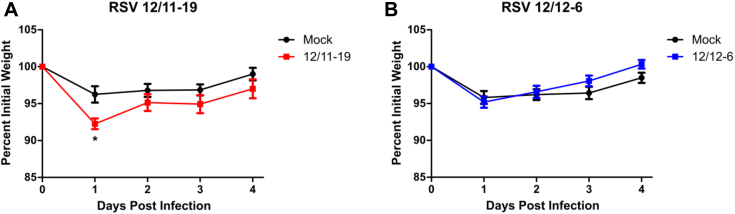
RSV-induced weight loss with 2012 clinical isolates. WT mice were infected with RSV strains 12/11-19 (1.0 × 10^6^ PFU; **A**) or 12/12-6 (9.0 × 10^5^ PFU; **B**), and weight loss was measured daily for 4 days. Data are displayed as a percentage of initial body weight and represent means ± SEMs. For Fig E9, *A-B*, n = 8-10 mice per group from 2 independent experiments. *P < .05, 2-way ANOVA.

**Fig 5:**
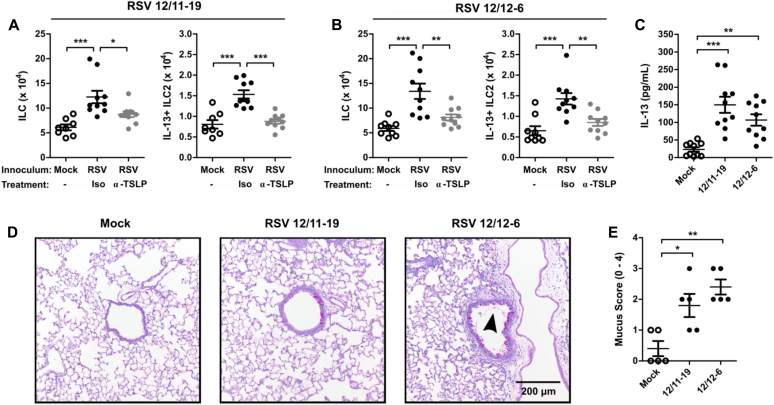
Pathogenic clinical isolates of RSV induce IL-13^+^ ILC2s through TSLP. WT mice were infected with RSV strains 12/11-19 (1.0 × 10^6^ PFU) or 12/12-6 (9.0 × 10^5^ PFU), treated with either 200 μg of isotype or anti-TSLP antibody at 6 hours after infection, and harvested on day 4 or 6 after infection. **A** and **B,** Total numbers of ILCs and IL-13^+^ ILC2s in mice infected with RSV 12/11-19 (Fig 5, *A*) and RSV 12/12-6 (Fig 5, *B*) at day 4 after infection. **C,** Whole-lung IL-13 levels measured by means of ELISA (right and left lung) at day 4 after infection. **D,** Representative PAS-stained sections of mucus-containing airways in the lungs on day 6 after infection (×20 magnification). The *arrowhead* denotes intraluminal airway mucus. **E,** Quantification of airway mucus from the experiment in Fig 5, *D*. Each *dot* represents a combined airway mucus score for an individual mouse. Data are plotted as means ± SEMs. For Fig 5, *A* and *B*, n = 8-10 mice per group combined from 2 independent experiments. For Fig 5, *C*, n = 10 mice per group combined from 2 independent experiments. For Fig 5, *D* and *E*, n = 5 mice per group. *P < .05, **P < .01, and ***P < .001, 1-way ANOVA.

To determine the generalizability of our results and their clinical relevance to strains of RSV with known human pathogenic potential, we evaluated the ability of 2 recently collected clinical isolates of RSV to induce ILC and IL-13^+^ ILC2 numbers and the necessity of TSLP in this process. RSV strains 12/11-19 and 12/12-6 were both isolated in 2012 as part of the INSPIRE study from 2 different patients who were hospitalized with severe lower respiratory tract infection and bronchiolitis.^28^ Mice were infected with 1.0 × 10^6^ or 9.0 × 10^5^ PFU of 12/11-19 or 12/12-6, respectively, and the number of ILCs and IL-13^+^ ILC2s was determined at day 4 after infection. Consistent with our results obtained with RSV strain 01/2-20, both 12/11-19 and 12/12-6 induced a significant expansion of total ILCs and IL-13^+^ ILC2s by day 4 after infection (Fig 5, *A* and *B*). Moreover, anti-TSLP neutralizing antibody was able to significantly attenuate the RSV-induced IL-13^+^ ILC2 response for both 12/11-19 and 12/12-6 (Fig 5, *A* and *B*). Importantly, both of these clinical isolates induced the accumulation of IL-13 in the whole-lung homogenate at day 4 after infection and airway mucus at 6 after infection (Fig 5, *C-E*), although significant weight loss was only observed with 12/11-19 (see Fig E9 in this article's Online Repository at www.jacionline.org). Collectively, these data demonstrate that the TSLP-dependent activation of ILC2s is a conserved feature among RSV strains with known human pathogenic potential that have recently circulated in the human population.

## Discussion

The immunologic contributions to the pathophysiology of severe RSV infection are incompletely understood. Our studies implicate the recently described ILC2s as a key source of IL-13 during the early stages of RSV infection in a murine model. We demonstrated that TSLP signaling was required for induction of ILC2s during RSV infection. Moreover, TSLPR-deficient mice had reduced airway mucus, airway reactivity, and weight loss and a similar viral load compared with WT mice, suggesting that TSLP might be a potential therapeutic target for IL-13–driven immunopathology associated with RSV. Finally, we identified that multiple recently collected clinical isolates of RSV with known human pathogenic potential induced IL-13–producing ILC2s through a TSLP-dependent mechanism. Collectively, these data demonstrate the importance of ILC2s and TSLP during the early stages of RSV infection and are the first to link TSLP to the activation of ILC2s during a viral respiratory tract infection.

TSLP is primarily produced by epithelial cells, especially those in the lungs and gut, as well as keratinocytes in the skin.^42^, ^43^ Lung epithelial cells are the primary site of RSV infection in human subjects and mice,^9^, ^27^ suggesting a means by which TSLP can be elaborated rapidly after infection. Interestingly, direct treatment of purified lung ILCs with TSLP alone did not induce cell proliferation or IL-5 and IL-13 cytokine production (data not shown; all values were less than the limit of detection). It is possible that TSLP is acting on non-ILCs to produce further signals that activate ILC2s. However, our data demonstrating that lung ILC2s express the TSLPR and that changes in TSLPR expression are restricted to the ILC2 subset, which is increasing in number, suggest that TSLP is likely having direct effects on ILC2s. Previous studies have shown that TSLP acts synergistically with other stimuli, rather than individually, to potentiate murine and human ILC2.^44^, ^45^ Together, these data suggest that an additional collaborative partner or partners might play a role in conjunction with TSLP during the *in vivo* activation of ILC2s after RSV infection.

IL-33 has been shown to be an important activator of ILC2s in mouse models of allergic airway inflammation, influenza infection, and rhinovirus infection.^12^, ^14^, ^16^, ^18^, ^19^, ^21^, ^46^, ^47^ Interestingly, we found similar numbers of IL-13–producing ILC2s after RSV infection in IL-33–deficient mice, although there was a statistically significant decrease in the total lung concentration of IL-13 at day 4 after infection. These incongruous results could be explained in the IL-33–deficient mice by potentially defective functionality of ILC2s despite similar numbers as seen in WT mice or different kinetics or localization of the ILC2 response. Also, we cannot exclude additional sources of IL-13 in these mice, including epithelial cells themselves,^48^ which could be susceptible to IL-33 deficiency, leading to decreased total lung IL-13 concentrations despite stable numbers of IL-13–producing ILC2s. Age-variable effects of IL-33 on ILC2s in the mouse model of RSV infection might also play a role.^49^

Furthermore, ILC2 activation in the neonatal mouse model of rhinovirus infection requires IL-25.^17^ Additionally, RSV strain Line 19 has been shown to induce IL-25 transcription in mice.^41^ However, we did not detect this cytokine by using ELISA after RSV infection with strain 01/2-20, suggesting it is less likely to play a role in this model.

Beyond IL-33 and IL-25, additional proinflammatory molecules, including cysteinyl leukotrienes (notably leukotriene D_4_) and TNF family cytokines (notably TLA1) have recently been identified as activators of ILC2s and might play a role, in conjunction with TSLP, during RSV infection.^50^, ^51^ Future studies will be required to precisely define the network of cytokines influencing ILC2 activation during RSV infection, although our data strongly suggest a critical role for TSLP in this milieu.

Importantly, the identification of TSLP as an activator of ILC2s is distinct from other respiratory tract viruses. These data highlight that different respiratory tract viruses induce ILC2s through unique mechanisms. Determination of the precise stimuli for ILC2s in each disease model will be necessary when conceptualizing and developing therapeutics, specifically with monotherapies targeting only IL-33, IL-25, or TSLP.

A recent phase I randomized controlled trial evaluated the effectiveness of an anti-TSLP mAb, AMG 157, in reducing disease severity in patients with mild allergic asthma.^52^ Patients randomized to AMG 157 had reduced airway obstruction and inflammation relative to patients receiving placebo after aeroallergen challenge. Both airway obstruction and inflammation are associated with T_H_2 cytokines, including IL-13. Given that RSV induces a TSLP-dependent, IL-13–producing ILC2 response in mice and that IL-13 promotes airway obstruction, it is intriguing to consider the therapeutic potential of an anti-TSLP mAb for treating severe RSV infection. Our studies demonstrate that a neutralizing mAb targeting TSLP was capable of reducing the number of IL-13–producing ILC2s in the lungs of mice after RSV infection, providing a small-animal model proof of concept for this therapeutic approach. Critically, it has been demonstrated that RSV infection can stimulate the production of TSLP in primary human airway epithelial cells, supporting the translational significance of TSLP during RSV infection.^39^ In addition, TSLP has previously been shown to enhance the CD4^+^ T_H_2 compartment during the later stages of RSV infection.^39^, ^40^ The use of an anti-TSLP mAb might also have the dual benefit of decreasing both ILC2 and CD4^+^ T_H_2 contributions to immunopathology. Additional studies will be required to further assess the feasibility of an anti-TSLP mAb during severe RSV infection in human subjects.

Interestingly, neutralization of TSLP significantly attenuated IL-13^+^ ILC2 induction during RSV infection irrespective of whether intervention occurred 6 or 36 hours after infection. This was surprising given that the peak of TSLP in the lungs occurred 12 hours after infection. One potential explanation for these data is the continued production of TSLP in the lungs beyond 12 hours after infection, which is physiologically significant but less than the limit of detection of the ELISA. Dynamic changes in TSLPR expression on ILC2s might play a critical role in responsiveness to this submaximal TSLP. Specifically, we identified that TSLPR is increased in expression on ILCs after RSV infection and that the increased expression clusters most significantly with proliferating Ki67^+^IL-5^−^IL-13^+^ ILC2s. Increased receptor expression in this subset might allow for continued responsiveness to levels of TSLP that are less than the limit of detection of our ELISA.

Furthermore, our data suggest a deficiency in TSLP signaling is unlikely to affect viral load and might improve clinical illness as measured by weight loss, all while attenuating IL-13–producing ILC2s, whole-lung concentrations of IL-13, airway mucus accumulation, and airway reactivity. Although the human pathogenesis of RSV is multifaceted, inclusive of both viral and host contributions, several lines of evidence suggest that immunopathology and airway mucus hypersecretion promote disease severity in infants.^9^, ^53^ Importantly, we did not identify any significant increase in viral load in TSLPR-deficient mice, suggesting that the targeting of the TSLP axis is unlikely to exacerbate virally associated disease metrics while still providing relief to immunopathologic changes in the airways. It is relevant to note that although weight loss is a common measure of illness severity in mice, the direct implications of this murine observation to prognosis or disease course in human subjects remain unclear.

Viral respiratory tract infections, including RSV, are an important cause of asthma exacerbations in both children and adults.^54^ Atopic asthma is associated with increased levels of T_H_2 cytokines in the airways, including IL-4.^55^, ^56^, ^57^ Several studies demonstrate that IL-4 enhances the capacity of human airway epithelial cells to produce TSLP.^58^, ^59^ Moreover, the combination of IL-4 plus double-stranded RNA synergistically enhanced production of TSLP from human airway epithelial cells.^58^ Together, it is intriguing to consider whether a mechanism exists for RSV-induced asthma exacerbation whereby pre-existing allergic inflammation and IL-4 production can prime airway epithelial cells to produce augmented levels of TSLP on infection with RSV, leading to an exaggerated ILC2 response with increased T_H_2 cytokine production. Consistent with this hypothesis, airway epithelial cells isolated from asthmatic children express greater levels of TSLP on *in vitro* RSV infection than airway epithelial cells isolated from nonasthmatic children.^39^ Further studies will be needed to determine whether such a mechanism exists and, if so, to what degree it influences RSV-exacerbated asthma.

Our data demonstrate that ILC2s are an important source of IL-13 during the early stages of RSV infection in a murine model. This process required TSLP signaling, and the lack of TSLP signaling did not negatively affect viral load but significantly reduced disease severity, as measured by weight loss, airway mucus accumulation, and airway reactivity. Additionally, infection of mice with 2 recent clinical isolates of RSV with known human pathogenic potential similarly induced IL-13–producing ILC2s through a TSLP-dependent mechanism. These studies have significant and broad implications for the targeting of ILC2s during primary RSV infection, potentially through neutralization of TSLP, as well as during RSV-induced wheezing illnesses.

**Key messages**

• RSV infection increased the number of IL-13–producing ILC2s through TSLP signaling in a murine model of RSV infection.
• Deficiency in TSLP signaling decreased lung IL-13 concentrations, attenuated airway mucus and reactivity, did not enhance viral load, and partially protected against RSV-induced weight loss.
• Multiple RSV strains isolated from patients hospitalized with severe RSV-induced bronchiolitis were capable of inducing IL-13–producing ILC2s through TSLP in a murine model.

## Methods

### Virus and mice

Unless otherwise noted, all experiments were performed with RSV strain 01/2-20, which was isolated from a patient in 2001 in the Vanderbilt Vaccine Clinic (Nashville, Tenn).^E1^ The 01/2-20 strain was selected because of its recent isolation with limited *in vitro* passaging and its mucogenic potential. Where indicated, we performed experiments using RSV strains 12/11-19 and 12/12-6, which were isolated in 2012 from hospitalized patients with severe lower respiratory tract infection and bronchiolitis as part of the INSPIRE study.^E2^ These viruses were propagated and titrated in HEp-2 cells, as previously described.^E3^ Mock inoculum was prepared by collecting cell-culture supernatants from lysed uninfected HEp-2 cells. UV inactivation was performed by exposing the virus directly to a 30-W UV light for 40 minutes on ice. Female, 8- to 12-week-old IL-33 citrine reporter (IL-33-deficient; IL-33 KO) mice, TSLPR-deficient (TSLPR KO) mice, or WT BALB/c (WT) mice were used in compliance with the revised 1996 “Guide for the care and use of laboratory animals” prepared by the Committee on Care and Use of Laboratory Animals of the Institute of Laboratory Animal Resources, National Research Council. IL-33 KO mice and TSLPR KO mice were generated on or backcrossed to a BALB/c background, as previously described.E4, E5, E6 WT BALB/c mice were purchased from the Jackson Laboratory. Mice were housed in microisolator cages under specific pathogen-free conditions. For infection, mice were anesthetized with a ketamine/xylazine solution and inoculated by means of intranasal delivery with 3.0 × 10^6^ PFU of RSV 01/2-20, 1.0 × 10^6^ PFU of RSV 12/11-19, 9.0 × 10^5^ PFU of RSV 12/12-6, or an equal volume of mock inoculum, as previously described.^E3^ Illness severity was measured daily based on weight loss.

### ELISA

Lungs were snap-frozen in liquid nitrogen at the time of harvest. Lungs were mechanically disrupted by using 1 mL of RIPA buffer (Sigma-Aldrich, St Louis, Mo) with complete protease inhibitor (Roche Applied Science, Penzberg, Germany) in a BeadBeater (BioSpec Products, Bartlesville, Okla). Protein measurements were performed, according to the manufacturer's instructions, by using ELISA kits from R&D Systems for IL-25 (DuoSet), IL-33 (DuoSet), TSLP (Quantikine), IL-4 (Quantikine), IL-5 (Quantikine), and IL-13 (Quantikine).

### Flow cytometry

Right and left lungs were digested in RPMI media with 5% FBS, 1 mg/mL collagenase, and 0.02 mg/mL DNase I for 60 minutes at 37°C. A single-cell suspension was generated by straining these digestions through a 70-μm filter. RBC lysis (BioLegend, San Diego, Calif) was performed, according to the manufacturer's instructions. Cells were restimulated with 10 ng/mL phorbol 12-myristate 13-acetate and 1 μmol/L ionomycin in the presence of 0.07% monensin in Iscove modified Dulbecco medium media supplemented with 10% FBS, 0.01 mmol/L nonessential amino acids, penicillin/streptomycin, and 1 mmol/L sodium pyruvate for 6 hours at 37°C. Cells were stained with LIVE/DEAD Blue (Life Technologies, Carlsbad, Calif) and combinations of the following surface markers: CD45 (30-F11) and CD25 (PC61.5) from eBioscience (San Diego, Calif); CD127 (SB/199) and CD3 (17A2) from BioLegend; TSLPR (goat polyclonal) from R&D Systems; and a surface marker cocktail containing CD5, CD45R (B220), CD11b, Gr-1 (Ly-6G/C), 7-4, and Ter-119 from Miltenyi Biotech (Bergisch Gladbach, Germany).

Cells were fixed/permeabilized and stained with combinations of the following intracellular markers: IL-13 (eBio13A) and Ki67 (SolA15) from eBioscience and IL-5 (TREK5) from BD Biosciences. All samples were run on a BD LSR II Flow Cytometer and analyzed with FlowJo Software Version 10.

Total ILCs were defined as Lin^−^CD45^+^CD25^+^CD127^+^ cells, where Lin includes CD3, CD5, CD45R (B220), CD11b, Gr-1 (Ly-6G/C), 7-4, and Ter-119. ILC2s were defined as ILCs that expressed IL-5, IL-13, or both. T cells were defined as CD45^+^CD3^+^ cells. MFI was determined as the geometric mean.

### *In vivo* TSLP neutralization

The monoclonal hybridoma cell line 28F12 was grown, and anti-TSLP antibodies were purified by the Vanderbilt Antibody and Protein Resource. The 28F12 hybridoma was developed by Dr Andrew Farr and obtained from the Developmental Studies Hybridoma Bank. The Developmental Studies Hybridoma Bank was created by the Eunice Kennedy Shriver National Institute of Child Health and Human Development of the National Institutes of Health and maintained at the University of Iowa, Department of Biology, Iowa City, Iowa. The 28F12 mAb has demonstrated TSLP neutralizing activity with efficacy for *in vivo* depletion.E7, E8, E9 Purified rat IgG2aκ isotype control mAb was purchased from BioLegend (catalog no. 400544). At either 6 or 36 hours after RSV infection, mice received a single dose of 200 μg of 28F12 or isotype control antibody by means of intraperitoneal injection.

### BAL and PAS staining

An intratracheal tube was inserted, and lungs were flushed with 0.8 mL of physiologic saline solution. An aliquot of BAL fluid was taken to determine total cells counts. For cell differentials, 0.1 mL of BAL fluid was spun onto slides and stained with Diff-Quik kit (American Scientific Products, McGaw Park, Ill) to visualize macrophages, lymphocytes, eosinophils, and neutrophils. For PAS staining, lungs were perfused with 1× PBS, inflated with 10% neutral buffered formalin, and fixed in 20 mL of 10% neutral buffered formalin for 24 hours at room temperature. Lungs were then transferred to 70% ethanol, paraffin embedded, sectioned (5 μm), and stained with PAS to visualize mucus. Small- and medium-sized airways were scored for mucus by a trained pathologist blinded to the experimental information using the following scoring scheme as follows: 0, no PAS^+^ cells observed in cross-sections of medium to small airways; 1, less than 10 PAS^+^ cells observed in cross-sections of medium to small airways; 2, greater than 10 PAS^+^ cells observed in cross-sections of medium to small airways; 3, greater than 10 PAS^+^ cells observed in cross-sections of medium to small airways with mucous strands observed in air spaces; or 4, greater than 10 PAS^+^ cells observed in cross-sections of medium to small airways with mucous plugging of airways.

### Corticosteroid treatment

Dexamethasone 21-phosphate disodium salt (Sigma-Aldrich) was prepared by dissolving in PBS. Mice were treated with 2.5 mg/kg dexamethasone or an equivalent volume of PBS vehicle administered by means of intraperitoneal injection. Mice were treated 24 hours before infection, 1 hour before infection, and every 24 hours after infection until mice were killed.

### Airway reactivity

Airway reactivity was measured, as previously described.E10, E11 Briefly, mice were anesthetized with an intraperitoneal injection of pentobarbital sodium (85 mg/kg). A tracheostomy tube was inserted for ventilation. The internal jugular vein was cannulated for intravenous delivery of acetyl-β-methacholine. Mice were then placed in a whole-body plethysmography chamber and mechanically ventilated. Precision glass microsyringes were used to deliver increasing doses of acetyl-β-methacholine. Baseline airway resistance measurements were collected, followed by measurements with 45, 137, 411, and 1233 μg/kg body weight of acetyl-β-methacholine. Peak airway reactivity measurements for each dose were recorded.

### Viral load

Mice were infected with 3.0 × 10^6^ PFU of RSV 01/2-20, as described above. On days 2, 4, and 6 after infection, lungs were collected, weighed, and snap-frozen. Thawed lungs were resuspended in 1 mL of sterile MEM media and homogenized through a BeadBeater. Lung homogenates were serially diluted and used to infect subconfluent HEp-2 cells in 12-well plates for 1 hour at room temperature while shaking. Cells were subsequently overlaid with MEM supplemented with 10% FBS, penicillin G, streptomycin, gentamicin, amphotericin B, and 0.75% methylcellulose and incubated at 37°C. After 6 days, cells were fixed for 1 hour with 10% neutral buffered formalin and stained with hematoxylin and eosin to visualize plaques.

### Statistical analysis

Data were analyzed with GraphPad Prism Version 5. Differences between groups were evaluated by using the unpaired *t* test, 1-way ANOVA with the Bonferroni posttest, or 2-way ANOVA with the Dunn multiple comparison test, as appropriate. Measurements of less than the limit of detection were assigned half of the value of the limit of detection to allow for statistical analyses.
